# A Hybrid Trust Model against Insider Packet Drop Attacks in Wireless Sensor Networks

**DOI:** 10.3390/s23094407

**Published:** 2023-04-30

**Authors:** Youngho Cho, Gang Qu

**Affiliations:** 1 Department of Defense Science (Computer Engineering and Cyberwarfare Major), Graduate School of Defense Management, Korea National Defense University, Nonsan 33021, Republic of Korea; 2 Department of Electrical and Computer Engineering and Institute of Systems Research, University of Maryland, College Park, MD 20742, USA

**Keywords:** insider packet drop attack, trust model, trust-based routing, wireless sensor networks

## Abstract

Quick and accurate detection of inside packet drop attackers is of critical importance to reduce the damage they can have on the network. Trust mechanisms have been widely used in wireless sensor networks for this purpose. However, existing trust models are not effective because they cannot distinguish between packet drops caused by an attack and those caused by normal network failure. We observe that insider packet drop attacks will cause more consecutive packet drops than a network abnormality. Therefore, we propose the use of consecutive packet drops to speed up the detection of inside packet drop attackers. In this article, we describe a new trust model based on consecutive drops and develop a hybrid trust mechanism to seamlessly integrate the new trust model with existing trust models. We perform extensive OPNET (Optimized Network Engineering Tool) simulations using a geographic greedy routing protocol to validate the effectiveness of our new model. The simulation results show that our hybrid trust model outperforms existing trust models for all types of inside packet drop attacks, not only in terms of detection speed and accuracy as it is designed for, but also in terms of other important network performance metrics, such as packet delivery rate, routing reliability, and energy efficiency.

## 1. Introduction

In wireless sensor networks (WSNs), sensor nodes will generate data packets and send them to the base station (BS) in a collaborative multi-hop fashion to overcome the problem of their limited energy and transmission range. While being routed to the BS, data packets may be lost because of collision, congestion, fading, obstacles in the field, or other network problems. The so-called *insider packet drop attacks* refer to a set of attacks where compromised nodes intentionally drop packets [1,2]. These attacks have become a serious security threat in WSNs [1,3,4,5,6,7]. A well-positioned attacker can be on the routing path of many nodes and thus receive many data packets. It can simply drop them to cause damage to the network. Moreover, it is challenging to detect and defend against such inside attackers because they can disguise their malicious packet dropping behavior behind the aforementioned natural packet loss phenomenon.

A trust mechanism has been proven as a promising approach to identify inside packet drop attackers [8,9,10,11,12,13,14,15]. In this approach, each node will monitor its neighbor’s packet forwarding behavior and use this observation to measure the trustworthiness of its neighbors. Once a neighbor’s trust value falls below a pre-determined threshold, the monitoring node will consider this neighbor as an inside attacker, eliminate it from the routing table, and establish a new routing path to avoid it. Such trust-based routing approaches can gracefully mitigate insider packet drop attacks by building trustworthy paths to the BS. It is shown that trust-based routing can improve the packet’s successful delivery under insider packet drop attacks over routing algorithms that do not consider trust [8,11,12,14,16]. The simplicity and effectiveness make trust mechanisms a good choice to mitigate insider packet drop attacks in a WSN where the nodes have limited resources and computation power.

However, inside attackers are also legitimate members of the network and they will know the trust mechanism being used. As we will elaborate on later, it is important for attackers to launch insider packet drop attacks without being detected by the existing trust mechanisms. Meanwhile, inside attackers can continue to drop packets and cause damage to the network performance before they are detected and eliminated from the network. Therefore, quick and accurate detection of inside packet drop attackers is critical to minimize the damage to the network, especially for networks that carry out mission-critical applications. Clearly, any characteristics that can distinguish inside packet drop attackers from other normal nodes will help in evaluating a node’s trustworthiness and thus improves the detection effectiveness of trust mechanisms.

In this article, we propose *consecutive packet drops* as one of these characteristics for the following three reasons. First, in many applications, inside packet drop attackers have to drop packets consecutively to achieve their goal, and this has been demonstrated in previous studies [9,12,17]. Second, a large number of consecutive drops are unlikely to happen naturally at a normal node. Third, when a normal node drops packets consecutively due to a network problem, although it is not an attacker, it may be advantageous to eliminate this node from the routing path to improve network performance. Basically, the first two reasons indicate that consecutive packet drops can distinguish behavior between attackers and normal nodes; the last reason argues that a false alarm (that is, misclassifying a normal node as an attacker) may not cause much damage to the network performance.

Current trust models, including the popular beta trust model [18] and entropy trust model [8], only consider the total number of packet drops and do not address the distribution of these drops. As we will show later in this article, such models cannot be effective in detecting inside attackers. To the best of our knowledge, this is the first attempt to build trust models based on packet dropping properties other than the total number. Our trust model takes consecutive packet drops into consideration and can be seamlessly integrated into existing models.

To show that evaluating the trust value of a node based on its consecutive packet drops can effectively improve the detection speed and accuracy, we propose a new trust model based on the concept of consecutive drops and then build a hybrid trust model that can adaptively choose between this new trust model and an existing trust model. We conduct extensive simulations using a commercially available network simulator, OPNET Modeler [19], to demonstrate that our model outperforms the beta trust model for all types of inside packet drop attacks, not only in terms of detection speed and accuracy as it is designed for, but also in terms of other important network performance metrics, such as packet delivery rate, routing reliability, and energy efficiency.

The rest of the paper is organized as follows. We first describe the background in Section 2. In Section 3, we give the motivation for considering consecutive packet drops in evaluating trust in order to quickly and accurately detect inside packet drop attackers. In Section 4, we elaborate our hybrid trust model based on consecutive drops. In Section 5, we evaluate the performance of our model. Finally, we conclude in Section 6.

## 2. Background

### 2.1. Insider Packet Drop Attacks and Countermeasures

Inside attackers can launch various types of attacks actively (such as modification, packet drop, or misrouting) or passively (such as eavesdropping). Among these, insider packet drop attacks are the most challenging to defend because they cannot be prevented by authentication and authorization [1,3], and hence they can potentially cause significant degradation to the network performance. Below are three representative types of insider packet drop attacks [9,14,17]:

(1) Blackhole attack: This attacker will drop all the received packets. It causes the most serious damage to the network among all types of packet drop attacks. However, the monitoring neighbors can easily capture this attacker as it consistently drops all packets.
(2) On-off attack: This attacker will drop all received packets when it is in attacking mode and forward all the received packets when attack is off. It repeats this drop-forward pattern periodically. This attacker can be suspicious to its monitoring neighbors during its attack period when it acts like blackhole attacker, and thus can be detected easily when the attack period is long or the on-off pattern is discovered.
(3) Grayhole attack (selective forwarding attack): This attacker will drop some of the received packets, either randomly or selectively. For example, each time the attacker receives a packet, it may decide whether to drop it according to a predetermined attack rate (or drop rate). Another example is that the attacker may only drop packets of a specific type or those generated by a specific source node.

As described above, it is easy to catch blackhole attackers and there are also some relatively effective means to detect on-off attackers. The grayhole attackers remain the most difficult to detect because most of the time their behaviors are very similar to those of the normal nodes. Existing mitigation approaches against grayhole attackers are either detection-based or avoidance-based approaches.

Most of the detection-based approaches focus on random selective forwarding attacks [20,21,22,23]. For example, in the multi-hop acknowledgment scheme [20], each node in the forwarding path is responsible for detecting attackers. Specifically, some randomly chosen nodes (called ACK nodes) will report ACKs back to the source node (hop by hop) using the same but reversed routing path when they receive a packet. However, this approach has several problems. First, it is unclear how to locate the exact attacker. Second, their detection scheme depends on other nodes’ observations, and thus is vulnerable to false accusation from malicious neighbors. The trust mechanisms with watchdogs, on which we will elaborate in the next section, solve these two problems by monitoring whether the next node in the routing path forwards the packets or not [4,8,11,12].

Instead of detecting the attackers, the avoidance approaches focus on how to deliver the packets successfully with the existence of the attackers. A popular way to achieve this is to use multipath routing paths [3,24]. In [3], the authors pointed out that k disjoint multipath routing can completely defend against selective forwarding attacks with no more than *k* − 1 compromised nodes. However, the multipath routing approach has a couple of drawbacks [20]. First, communication overhead increases significantly as the number of paths increases, and thus it may lead to more collision and interference. As a result, the packet delivery performance of a routing can be dramatically degraded. Second, since this approach cannot catch and discard the attackers and this approach can be compromised if an adversary locates at least one attacker in each routing path. Similarly, a multiple data flow scheme using multiple disjoint topologies was introduced in [24].

### 2.2. Trust Mechanism

A trust mechanism defines a trust value (or trustworthiness) for each node and how each node measures the trustworthiness of its neighbors. It detects insider packet drop attacks in three stages: neighbor behavior monitoring, trust measurement, and attacker detection.

(1) Neighbor behavior monitoring: Each node monitors and records its neighbors’ behaviors, such as packet forwarding. Watchdog [4] is a popular monitoring mechanism used in this stage. Each node A records all recently forwarded packets in a buffer. When A sends a packet to its neighbor node B, A monitors whether B forwards the packet toward the BS by overhearing B’s packet transmission. Each overheard packet will be compared with the packets that A has sent to B. When A finds a match in its buffer, A will record that B has forwarded the packet and will remove it from the buffer. If a packet remains in the buffer for a period longer than a pre-determined time, the watchdog considers that B failed to forward the packet.
(2) Trust measurement: Based on the data collected in the previous stage, a trust model will measure the trustworthiness of the node being monitored [8,9,13,14,18,25,26,27]. For example, when a node is observed to have forwarded the packet *s* times and dropped the packet *f* times, the beta trust model [18] will assign this node a trust value using the following equation:

(1) $$T_{Beta}\left(s,f\right)=E\left[beta\left(p|s+1,f+1\right)\right]=\frac{s+1}{s+f+2}$$

where E[beta(*p*|*s* + 1, *f* + 1)] is the expectation of the probability density function of the beta distribution given s and *f* [9,28].

The entropy trust model [8] uses entropy function:

(2) $$H\left(p\right)=-p\log_{2}p-\left(1-p\right)\log_{2}(1-p)$$

where *p* is the trust value in the beta trust model, and define the trust value by:

(3) $$T_{Entr}=\left\{\begin{array}{cc} 1-H\left(p\right), & for\;0.5\leq p\leq 1;\\ H\left(p\right)-1, & for\;0\leq p<0.5. \end{array}\right.$$

Note that in Equation (1), the trust value is between zero and one. However, the trust value in Equation (3) is between −1 and 1. To have a non-negative trust value between zero and one, we define:

(4) $$T^{*}{}_{Entr}=\frac{1+T_{Entr}}{2}.$$

(3) Detection: By comparing the measured trust value with a pre-determined threshold *TH*, a node can decide whether its neighbor is trustworthy. If the neighbor’s trust value becomes lower than *TH*, it will consider this neighbor as an inside attacker. That is, if node A evaluates its neighbor B’s trust value as *T* and the trust threshold *TH*, the A will classify the state of trustworthiness of B, *S*[*B*], by the following simple comparison:

(5) $$S\left[B\right]=\left\{\begin{array}{cc} Trustworthy & if\;T\geq TH\\ Untrustworthy & if\;T<TH \end{array}\right.$$

Since inside packet drop attackers are likely to have low trust values, they will be classified into untrustworthy nodes and discarded from the monitoring node’s routing table. Depending on the network’s trust mechanism, the detection of inside packet drop attackers may or may not be broadcast to the rest of the nodes in the network. In our paper, we assume that the decision will not be broadcast for simplicity.

### 2.3. Trust-Based Routing

Once an inside attacker is detected, a node normally will find a different route to avoid the attacker. We use the popular greedy perimeter stateless routing (GPSR) [29] as an example to show how a trust mechanism can help to detect and avoid inside packet drop attackers.

**Figure 1:**
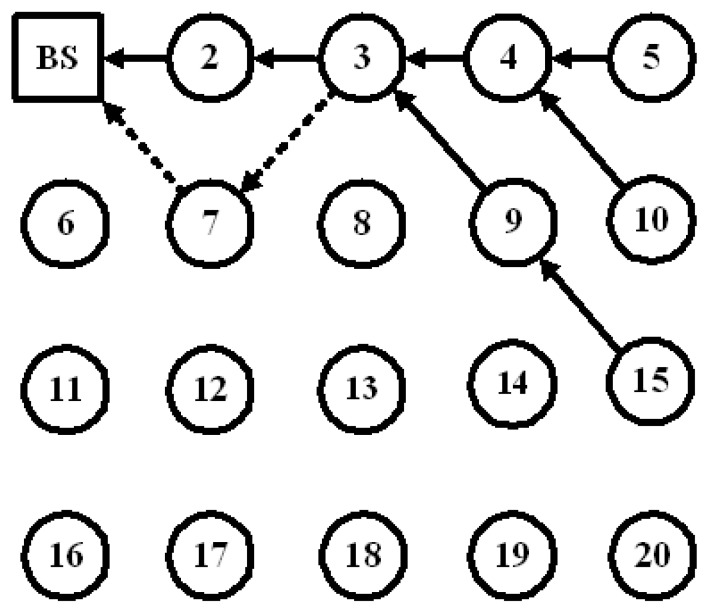
GPSR (solid lines) and trust-based GPSR (dotted lines); the numbers in circles represent sensor nodes’ IDs.

Consider a network with 20 nodes as shown in Figure 1. Node 3 relays its packets as well as those from nodes 4, 5, 9, 10, and 15 to node 2, which will then send them to the BS based on GPSR (depicted by solid lines with arrowhead). In a trust mechanism, node 3 will use its watchdog to monitor node 2.

When node 2 drops packets from node 3, node 3 will re-evaluate the trust value of node 2. If the trust value falls below the trust threshold value, node 3 will treat node 2 as an inside attacker. A trust-based routing algorithm will then find a new routing path to avoid node 2. In this case, node 3 will forward the packets to node 7, hoping that node 7 will deliver the packets to the BS (as shown by the dotted lines with arrowheads in Figure 1).

## 3. Motivation of the Proposed Work

Vulnerabilities exist in each of the three stages of the trust mechanisms. As the limitations of watchdogs have been well-documented [4,30], we will focus on the last two stages to motivate our work in this article.

First, in the trust measurement stage, the penalty on packet drops by current trust models does not effectively punish the attacker. It results in a long detection time to identify the attack during which damage will be made, particularly when the attackers have previously earned high trust value (called traitors). For example, when 0.80 is used as the trust threshold, an attack with initial trust value 0.99 can drop 183 and 84 packets before being detected by the beta and entropy trust models, respectively (see Section 5 for more details).

Second, in the detection stage, the major threat comes from the fact that the inside attacker, as a legitimate member of the network, knows the network’s trust mechanism and the trust threshold *TH*. Therefore, the attacker can estimate its trust value based on its own packet forwarding and dropping behavior, and then decide whether it would attack accordingly. For instance, when the network uses 0.80 as the trust threshold, a grayhole attacker can drop slightly less than 20% of the packets it receives without being detected, and an on-off attacker can keep its attacking period to be less than 20% to avoid being identified.

We believe that the rationale behind the inefficiency of the current trust models is that they solely rely on the number of total failures and completely ignore the distribution of these packet delivery failures. As the first attempt to measure the trust value with information beyond the total number of successes and failures, we propose to use consecutive packet drops (or consecutive delivery failures) for the early detection of inside packet drop attackers. Detecting inside attackers as soon as possible (early detection) is critical to minimize the damage they may cause to the network because undetected attackers can repeatedly drop packets and thus degrade the network performance.

Our work is motivated by the following observation: consider the following two sequences of packet delivery information, where each contains 10 successes and 10 failures:

ffssfsfsfsfffsssfsfs

ssssssssssffffffffff

The latter sequence looks more suspicious to be an insider packet drop attack due to the last 10 consecutive drops. This is also supported by the practical assumption that inside attackers start attacks after they earn high trust values (e.g., the first 10 successes in the latter observation) to avoid being easily detected [31]. However, both the beta and entropy trust models will treat these two sequences equally and score the same trust value on them.

Furthermore, suppose that a node’s trust value is approximately 1 initially, for example, after it successfully forwarded 1000 packets (that is, *s* = 1000). Then, the node starts dropping packets one by one without forwarding any of them. As the number of consecutive packet drops *n* goes up, its trust value *T* will be reduced. For example, when *n* goes from 1 to 30, the trust values in the beta trust model and the entropy trust model are calculated by Equations (1) and (4), respectively. Surprisingly, after 30 consecutive drops (*n* = 30), the trust values in the beta trust model and the entropy trust model are 0.969 and 0.902, respectively, which means this attacker will not be detected unless we use very high trust thresholds.

The goal of this work is to enhance the current trust models so that they can (i) measure a node’s trust value more accurately and (ii) detect insider packet drop attacks more effectively.

## 4. Trust Model Based on Consecutive Drops

We propose the use of consecutive packet drops to detect inside packet attacks. In this section, we first explain the rationale behind this approach and then describe a new trust model based on the concept of consecutive drops. Finally, we show how this new model can be integrated into existing trust models.

### 4.1. Rationale behind Consecutive Packet Drops

According to the most popular packet drop attack models, the inside packet drop attackers either drop as many packets as possible to degrade network performance, such as packet delivery rate, or prevent some critical data packets from reaching the destination (Denial-of-Service attack) [32,33]. To achieve these goals, an attacker will need to generate frequent or long consecutive packet drops. Therefore, consecutive packet drops become a unique characteristic for insider packet drop attacks and can be used to distinguish inside attackers from good nodes. We will elaborate on this rationale next.

First, in both blackhole and on-off attack models, the attacker will have to drop packets consecutively according to the definitions of these attacks. For a grayhole attacker, if they are not allowed to drop packets consecutively, the maximum damage they can cause will be very limited. For example, the attacker cannot drop more than 50% of packets that it receives without dropping packets consecutively. Therefore, we conclude that consecutive packet drops will happen in all types of insider packet drop attacks and should be considered in the design of trust models and trust-based defense mechanisms.

Second, for many WSN applications, such as fire detection, enemy tracking, battlefield surveillance, and disaster prevention [34,35], inside attackers have to drop packets consecutively in order to achieve their malicious goal. In the case of DoS attacks where the attacker wants to block all packets from victim nodes from being delivered to the BS, we consider the example of a WSN deployed in a territory for intruder detection. With the help of insiders that perform this DoS attack, an intruder will be able to enter the territory from the area monitored by victim nodes. If the inside attacker does not drop packets consecutively, some packets with critical information about the intruder will reach the BS, and thus the BS can detect the intruder. In this intruder detection problem, consecutive drops are more critical than in the former case (network performance degradation).

Third, statistically, a large number of consecutive drops are much more likely to happen at attacking nodes than at nodes functioning normally in the network. We can probabilistically measure the degree of abnormality of a node generating consecutive drops as follows. Let *P*[*f*] be the probability that a packet is dropped at a normally functioning node. Then, the probability that a packet is forwarded successfully will be *P*[*s*] = 1 − *P*[*f*]. If we observe 10 consecutive drops at the node, then the probability that the 10 drops occur consecutively is *P*[*f*]^10^. When we assume *P*[*f*] < *P*[*s*] in a well-designed WSN, even if *P*[*f*] is 0.5, *P*[*f*]^10^ ≈ 0.000977(=0.977 × 10^−3^), which means that it is very unlikely that 10 consecutive drops are happening. As n grows, the probability that n consecutive drops happen (*P*[*f*]^n^) decreases exponentially. For a more realistic example, when *P*[*f*] = 0.05, the probability that a good node drops three packets in a row is 0.05^3^ ≈ 0.977 × 10^−6^. For an attack that drops 70% of its packets, this probability is 0.343, approximately 3.51 × 10^5^ higher than the above good node.

Therefore, we believe that consecutive packet drops will happen in all types of insider packet drop attacks but are very unlikely to happen at a good functioning node. This unique characteristic should be considered in the design of trust models and trust-based defense mechanisms against insider packet drop attacks. As the above analysis shows, three consecutive drops already indicate a significant abnormality. We do not need a very large number of consecutive packet drops to distinguish an attacker from a good node.

Finally, using consecutive drops for detecting packet drop attacks may generate a false alarm. That is, it may lead us to treat a good node (non-attacker) as an attacker. In practice, a node (non-attacker) may drop many packets consecutively due to fading or obstacles. Detecting these nodes as attackers is a false alarm. However, if a good node malfunctions and generates a large number of consecutive drops, we should consider this node untrustworthy and not use it to ensure the quality of packet delivery performance of a routing. So, such false alarms may be helpful. In fact, we will show that avoiding such problematic nodes quickly reduces the packet drop rate in Section 5.

### 4.2. Design of the New Trust Model

#### 4.2.1. New Trust Model

##### Properties against Inside Packet Drop Attacks

The new trust model must have the following two properties:

- Property 1: As the number of consecutive failures grows, we increase the trust-decreasing rate. Thus, while we observe consecutive failures, we give a larger penalty for a new failure than the previous failures, because as the size of consecutive failures grows, our confidence that the consecutive failures are occurring due to attacks also grows. This property not only helps to quickly detect an attacker dropping packets consecutively but also forces a rational inside attacker to stop launching as many consecutive drops in the fear of being caught by the trust model.
- Property 2: Given two nodes B and C, where node A trusts B more than C based on the same number of their past behaviors, if both B and C are generating the same number of consecutive failures, A loses C’s trust more than B’s trust. For example, consider the following case. B behaved 40 times well and behaved 20 times badly, and node C behaved 20 times well and behaved 40 times badly. Now both B and C are misbehaving 5 times consecutively. Intuitively, it is reasonable to lower C’s trust further than B, because C’s past behaviors are worse than node B. This property will work against grayhole attacks and on-off attacks.

##### Design of New Trust Function

We designed a new trust function based on the number of consecutive failures. Every trust model decreases a certain amount of trust value when a failure occurs. We considered the decreased amount of trust value as a penalty for the failure. We first designed a penalty function *PT*(*n*) that determines how much we should lower the trust value of a node that generates *n* consecutive failures. By using *PT*(*n*), given *n* consecutive failures, the new trust function *T*(*n*) was formulated as:

(6) $$T\left(n\right)=T\left(0\right)-PT\left(n\right)$$

where *T*(0) is the initial trust value of the evaluated node generating *n* consecutive failures at the evaluating node. Thus, when an evaluated node is generating *n* consecutive failures (*n* ≥ 1), we measure its trust value by using *T*(*n*) by deducting a certain amount of penalty *PT*(*n*) from *T*(0) which is its trust value just before it generates *n* consecutive failures.

We now explain how to determine the penalty for *n* consecutive failures. Consider *n* consecutive failures f_1_f_2_…f_i_…f_n_ whose *i*-th failure occurs at time *t* = *i* (1 ≤ *i* ≤ *n*). When *t* = 1, there is only one failure f_1_ and we assign *α* as a penalty to the single failure f_1_. Thus, *PT* (1) = *α*. When *t* = 2, another failure f_2_ occurs. Therefore, we assign *α* to the single failure f_2_. In addition, since we observe double failures f_1_f_2_ at *t* = 2, we give an extra penalty to f_1_f_2_. The probability that two consecutive failures occur is *P*[*f*]^2^ and our confidence that they should not occur is 1 − *P*[*f*]^2^. Since we give *α* as the penalty to a single failure when we have a confidence of 1 − *P*[*f*], by multiplying *α* by the ratio of confidence, we can get the penalty to the double failure as *α ×* (1 − *P*[*f*]^2^)/(1 − *P*[*f*]). Then, the total penalty to the two consecutive failures *PT*(2) is 2*α* + *α ×* (1 − *P*[*f*]^2^)/(1 − *P*[*f*]) where 2*α* is the penalty for two single failures and *α ×* (1 − *P*[*f*]^2^)/(1 − *P*[*f*]) is the penalty for one double failure.

By this approach, to get *PT* (*n*) we first must find all *unit failures,* such as single failure, double failure, triple failure, and *n*-tuple failure within *n* consecutive failures. Given *n* consecutive failures, there are *n* single failures, *n* − 1 double failures, *n* − 2 triple failures… and one *n*-tuple failure. Next, as shown in Table 1, we calculate *unit penalty* for each unit failure probabilistically by multiplying the base penalty *α* by the confidence ratio of the unit failures. By this manner, we formulate *PT* (*n*) as:

(7) $$\begin{array}{ll} PT\left(n\right)= & n\times \alpha \times 1+\left(n-1\right)\times \alpha \times (1-P\left[f{]}^{2}\right)/\left(1-P\left[f\right]\right)+\left(n-1\right)\times \alpha \times (1-P\left[f{]}^{2}\right)/\left(1-P\left[f\right]\right)+\ldots \\ & 2\times \alpha \times (1-P\left[f{]}^{n-1}\right)/\left(1-P\left[f\right]\right)+1\times \alpha \times (1-P\left[f{]}^{n}\right)/\left(1-P\left[f\right]\right)\\ & =\overset{n}{\underset{i=1}{\sum }}\left(n-i+1\right)\times \alpha \times (1-P\left[f{]}^{i}\right)/\left(1-P\left[f\right]\right) \end{array}$$

where *α* is a base penalty for a single failure.

By using Equations (6) and (7), we get *T* (*n*) as:

(8) $$T\left(n\right)=T\left(0\right)-\overset{n}{\underset{i=1}{\sum }}\left(n-i+1\right)\times \alpha \times (1-P\left[f{]}^{i}\right)/\left(1-P\left[f\right]\right)$$

where *P*[*f*] = 1 − *T*(0) since *T*(0) is the expectation value of *P*[*s*] when *n* = 0 in the beta trust model.

To reduce the computation complexity, we have:

**Theorem 1.**  *The trust function T(n) defined in Equation (6) satisfies:*

(9) $$T\left(n\right)=T\left(n-1\right)-PT_{R}\left(n\right);$$

(10) $$PT_{R}\left(n\right)=P\left[f\right]PT_{R}\left(n-1\right)+\alpha n\;.$$

**Proof.**  From Equations (7) and (8), we have:

$$\begin{array}{l} T\left(n\right)=T\left(0\right)-\overset{n}{\underset{i=1}{\sum }}\alpha \left(n-i+1\right)(1-P\left[f{]}^{i}\right)/\left(1-P\left[f\right]\right)\\ =T\left(0\right)-\overset{n}{\underset{i=1}{\sum }}\alpha \left(n-i\right)(1-P\left[f{]}^{i}\right)/\left(1-P\left[f\right]\right)+\overset{n}{\underset{i=1}{\sum }}\alpha (1-P\left[f{]}^{i}\right)/\left(1-P\left[f\right]\right)\\ =T\left(0\right)-\left\{\overset{n-1}{\underset{i=1}{\sum }}\alpha \left(n-i\right)(1-P\left[f{]}^{i}\right)/\left(1-P\left[f\right]\right)+\overset{n}{\underset{i=1}{\sum }}\alpha (1-P\left[f{]}^{i}\right)/\left(1-P\left[f\right]\right)\right\}\\ =\left\{T\left(0\right)-\overset{n-1}{\underset{i=1}{\sum }}\alpha \left(n-i\right)(1-P\left[f{]}^{i}\right)/\left(1-P\left[f\right]\right)\right\}-\alpha \overset{n}{\underset{i=1}{\sum }}(1-P\left[f{]}^{i}\right)/\left(1-P\left[f\right]\right)\\ =T\left(n-1\right)-\alpha \overset{n}{\underset{i=1}{\sum }}(1-P\left[f{]}^{i}\right)/\left(1-P\left[f\right]\right). \end{array}$$

Next, let $PT_{R}\left(n\right)=\alpha \times \sum_{i=1}^{n}(1-P\left[f{]}^{i}\right)/\left(1-P\left[f\right]\right)$. Then,

$$PT_{R}\left(n\right)=\alpha \overset{n}{\underset{i=1}{\sum }}(1-P\left[f{]}^{i}\right)/\left(1-P\left[f\right]\right)\;=\alpha \left\{1+\frac{1-P{\left[f\right]}^{2}}{1-P\left[f\right]}+\frac{1-P{\left[f\right]}^{3}}{1-P\left[f\right]}+\cdots +\frac{1-P{\left[f\right]}^{n}}{1-P\left[f\right]}\right\}\;=\alpha \left\{\begin{array}{c} 1+\left(1+P\left[f\right]\right)+(1+P\left[f\right]+P\left[f{]}^{2}\right)+\cdots \\ +(1+P\left[f\right]+\cdots +P\left[f{]}^{n-1}\right) \end{array}\right\}\;=\alpha \left[\begin{array}{c} n+P\left[f\right]\{1+\left(1+P\left[f\right]\right)+(1+P\left[f\right]+P\left[f{]}^{2}\right)\cdots \\ +(1+P\left[f\right]+\cdots +P\left[f{]}^{n-2}\right)\} \end{array}\right]\;=\alpha \left\{n+P\left[f\right]\overset{n-1}{\underset{i=1}{\sum }}(1-P\left[f{]}^{i}\right)/\left(1-P\left[f\right]\right)\right\}\;=P\left[f\right]\left\{\alpha \overset{n-1}{\underset{i=1}{\sum }}(1-P\left[f{]}^{i}\right)/\left(1-P\left[f\right]\right)\right\}+an\;=P\left[f\right]PT_{R}\left(n-1\right)+\alpha n.$$

□

##### Base Penalty *α*

Next, we need to determine the value of the base penalty *α*. For a single failure (*n* = 1), we assign the amount of penalty that the beta trust model gives for a single failure by which our new trust function works the same as the beta trust model. This approach helps to make it easy to design a hybrid trust model as we explained in Section 4.1. Thus, given the number of successes *s* and the number of failures *f*:

(11) $$\alpha_{n=1}=T_{Beta}\left(s,f\right)-T_{Beta}\left(s,f+1\right)=\frac{\left(s+1\right)}{\left(s+f+2\right)}-\frac{\left(s+1\right)}{\left\{\left(s+1)+(f+2\right)\right\}}=\frac{\left(s+1\right)}{\left(s+f+2\right)\left(s+f+3\right)}.$$

Meanwhile, as explained in Section 4.2.1, for consecutive failures (*n* ≥ 2) we use our new trust model that penalizes the node generating the consecutive failures. However, since *α_n=_*_1_ in Equation (11) is not inversely proportional to a node’s initial trust value *T*(0), and thus it does not satisfy *Property 2*, we use a different base penalty by dividing *α* by the initial trust value *T*(0) as:

(12) $$\alpha_{n\geq 2}=\frac{\alpha_{n=1}}{T\left(0\right)}=\frac{1}{s+f+3}.$$

By Equation (12), the base penalty of a node is adjusted according to the node’s initial trust value such that the base penalty of a node with lower trust is larger than that of a node with higher trust.

**Theorem 2.**  *The trust function defined above satisfies Property 1 and Property 2.*

**Proof.**  We prove *Property 1* by showing that *PT*(*n*) is an increasing function and for *n* ≥ 2, *PT*(*n*) − *PT*(*n* − 1) > *PT*(*n* − 1) − *PT*(*n* − 2).

According to Equation (7):

(13) $$PT\left(n\right)-PT\left(n-1\right)=\alpha \overset{n}{\underset{i=1}{\sum }}\frac{(1-P\left[f{]}^{i}\right)}{\left(1-P\left[f\right]\right)}.$$

(14) $$PT\left(n-1\right)-PT\left(n-2\right)=\alpha \overset{n-1}{\underset{i=1}{\sum }}\frac{(1-P\left[f{]}^{i}\right)}{\left(1-P\left[f\right]\right)}.$$

By Equations (13) and (14), (*PT*(*n*) − *PT*(*n* − 1)) − (*PT*(*n* − 1) − *PT*(*n* − 2)) = *α(*1 − *P*[*f*]*^n^)/(*1 − *P*[*f*]*)* > 0, since *α* > 0, *n* > 0 and 0 ≤ *P*[*f*] < 1. We assume *P*[*f*] ≠ 1. Thus, *PT*(*n*) satisfies *Property 1*. Next, to prove *Property 2*, let nodes 1 and 2 have the same number of past behaviors (*s*_1_, *f*_1_) and (*s*_2_, *f*_2_), respectively, where *s*_1_ + *f*_1_ = *s*_2_ + *f*_2_. Assume that node 1′s trust value is higher than node 2′s trust value, such that *s*_1_ ≥ *s*_2_ and *f*_1_ ≤ *f*_2_. They are generating *n* consecutive failures equally. Let *α_1_* and *α_2_* be base penalties of node 1 and node 2, respectively. Then, when *n* ≥ 2, by (12), *α_1_* = (*s*_1_ + *f*_1_ + 3)^−1^ and *α_2_* = (*s*_2_ + *f*_2_ + 3)^−1^, and thus *α_1_* = *α_2_* since *s*_1_ + *f*_1_ = *s*_2_ + *f*_2_. Given the same base penalty, since *P*[*f*] of node 1 is less than *P*[*f*] of node 2, according to Equations (7) and (10), the total penalty of node 2 is larger than that of node 1. Therefore, the trust function satisfies *Property 2*.

##### Example of New Trust Model and Consecutive Drops

**Figure 2:**
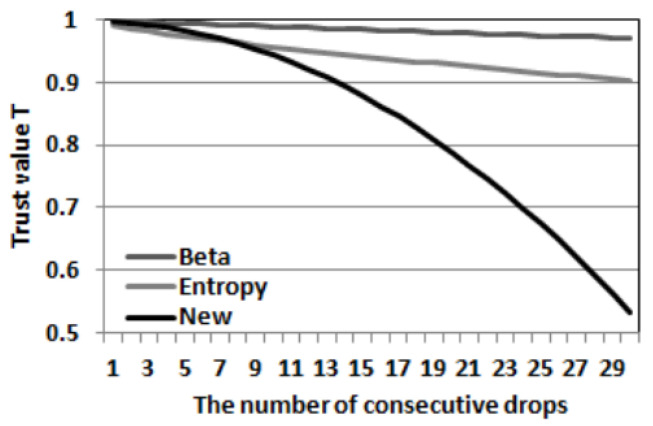
Trust value decreases as the number of consecutive packet drops increases according to beta, entropy, and the new trust models, respectively (from top to bottom).

We demonstrate how our model works by using the same example as in Section 3 and compare our trust model with the beta trust model and the entropy trust model. Again, a node’s trust value is approximately 1 after it successfully forwarded 1000 packets (that is, *s* = 1000), then the node starts dropping all received packets. As the number of consecutive drops *n* goes from 1 to 30, Figure 2 shows how their trust values *T* drop; the trust value in our new trust model is calculated by Equations (9) and (10). For the first failure (*n* = 1), our new trust model penalizes the node the same way as the beta trust model does. However, as *n* grows, our trust model gradually increases the penalty to the node (*Property 1*), and the trust value goes below 0.6 when *n* = 30. Assuming that the same trust threshold is used, we can see that our trust model detects inside packet drop attackers much faster than the beta trust model and the entropy trust model. In addition, if the node is an intelligent attacker that knows how our trust model works against its packet drops, to avoid being detected by our trust model, the attacker will not launch a long number of consecutive drops, which may lead to a failure in dropping consecutive critical packets completely, as discussed in Section 3. In the rest of our paper, we conduct extensive simulations based on the OPNET Modeler to show the performance of our trust model in practical network environments.

### 4.3. Design of Hybrid Trust Model

#### 4.3.1. Hybrid Trust Model Combining the Beta Trust Model with the New Trust Model

**Figure 3:**
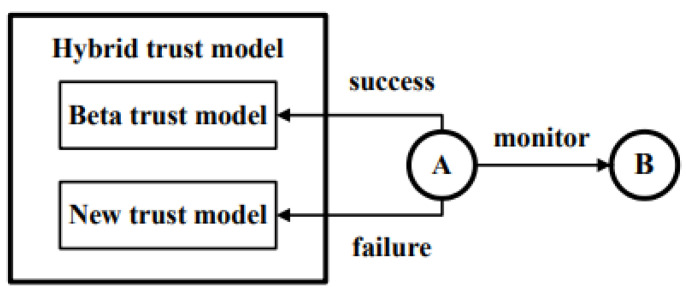
Hybrid trust model.

To show that trust evaluation using consecutive drops can effectively improve the early detection ability of a trust model, we propose a hybrid trust model that adaptively uses the popular the beta trust model and our new trust model based on consecutive drops. As shown in Figure 3, on each report of the behavior of a node B (that is, node A wants to evaluate the trust of node B), our hybrid trust model in node A works as follows:

- If the new behavior is a success, use beta trust model.
- If the new behavior is a failure, use new trust model based on the number of consecutive drops (or failures).

#### 4.3.2. State Transition between Two Trust Models

The hybrid trust model will adaptively choose either the beta trust model or the new trust model based on the occurrence of success or failure. As the two trust models use different formulas to evaluate trust value on the same input values of *s* and *f* (the total number of successes and failures, respectively), they may give different trust values. When we switch from the beta trust model to the new trust model on consecutive failures, we can use the current values of *s* and *f* to determine base penalty and then apply the new trust model to evaluate trust value. However, when we switch back to the beta trust model, the current trust value (from the new trust model) will be lower than the value based on the beta trust model (see Equation (1)). To continue using the beta trust model, we should adjust the parameters of the beta trust model so that the beta trust model has the same trust value as our new trust model. We call this procedure *state transition*.

One way to solve the state transition problem is to adjust the number of failures *f* in the beta trust model. Let *T_New_* and *T_Beta_* be the trust values in new trust model and the beta trust model, respectively. By *T_New_* = *T_Beta_*,

(15) $$T_{New}=\frac{s+1}{s+f^{*}+2}.$$

By Equation (15), we obtain the adjusted f* as:

(16) $$f^{*}=\frac{s+1}{T_{New}}-s-2.$$

## 5. Performance Evaluation

### 5.1. Goals, Metrics, and Methodology

#### 5.1.1. Goals of Experiments

Our main goal in this section is to show that our hybrid trust model that considers consecutive drops detects insider packet drop attacks more *quickly* and *accurately* than the beta trust model, which does not consider consecutive drops in the packet routing domain. In addition, since a routing algorithm must deliver packets to the destination *reliably* and *energy efficiently* even under insider packet drop attacks, we show how our hybrid trust model improves both the reliability and energy efficiency of a routing algorithm versus the beta trust model. We discuss reliability and energy efficiency in more detail in Section 5.1.4. We conduct extensive simulations based on the OPNET Modeler.

#### 5.1.2. Routing Algorithms for Performance Evaluation

For performance evaluation, we use the following three geographic greedy routing (GRP) algorithms:

- Geographic greedy routing without a trust model (Pure GRP);
- Pure GRP with the beta trust model (Beta GRP);
- Pure GRP with our model (Hybrid GRP).

The Pure GRP (geographic routing protocol) [19] provided by the OPNET Modeler is a geographic greedy routing system that works very similarly to the GPSR that we introduced in Section 2.1. We implemented two trust-based geographic greedy routings (beta GRP and hybrid GRP) based on the GRP and two trust models (the beta trust model and our hybrid trust model) in the OPNET Modeler.

The main difference between the three routings is the neighbor selection algorithm (NSA), by which a sending node S finds a next hop H to forward its packet to H. Algorithm 1 shows the neighbor selection algorithm of the Pure GRP. In the Pure GRP, as with the GPSR, a sending node S selects a next hop H that is the closest one to the destination from its neighbor set **NS**.
**Algorithm 1** (NSA in the GRP)Input: sender S, destination D, and S’s neighbor set **NS** Output: next hop node H1: d_MIN_: = dist (S, D); 2: **for** each node n_i_ in **NS do** 3:      calculate dist (n_i_, D); 4:      **if** (dist (n_i_, D) < d_MIN_) **then** 5:          H: = n_i_; 6:          d_MIN_: = dist (n_i_, D);

On the other hand, in the trust-based GRPs such as the Beta GRP or the Hybrid GRP, as shown in Algorithm 2, a sending node S finds a next hop H that is the closest one to the destination among its neighbors whose trust value is equal to or higher than the trust threshold TH. The only difference between the two trust-based GRPs is the difference in the trust models that they use.
**Algorithm 2** (NSA in the trust-based GRP)Input: sender S, destination D, and S’s neighbor set **NS,** trust threshold TH Output: next hop node H1: d_MIN_: = dist (S, D); 2: **for** each node n_i_ in **NS do** 3:      calculate dist (n_i_, D) and T (n_i_); /* *T(n_i_) is trust value of n_i_* */ 4:      **if** (d (n_i_, D) < d_MIN_ **and** T(n_i_) ≥ TH) **then** 5:          H: = n_i_; 6:          d_MIN_: = dist (n_i_, D);

#### 5.1.3. Packet Drop Attack Models

We used two types of packet drop attacks in simulations.

- Blackhole attack: drop all received data packets (attack rate is 100%).
- Grayhole attack: drop received data packets randomly according to various attack rates that range from 20% to 80%.

**Figure 4:**
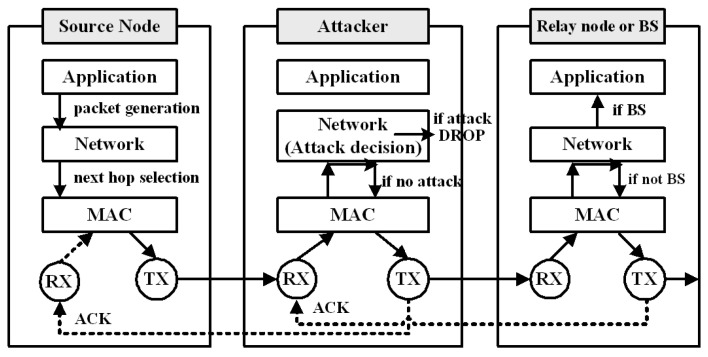
Implementation of packet drop attack models in the OPNET Modeler.

In our simulations, the attacker drops only data packets and forwards other types of packets, such as control packets. As shown in Figure 4, we implemented attack models at the network layer of a wireless node in the OPNET Modeler, since a node can determine whether a received packet is either a data packet or a control packet once the packet reaches the network layer. When an attacker receives a data packet from a node, the attacker sends an ACK message back to the node and then decides whether it drops a data packet based on a pre-defined attack rate. For example, if the attack rate is 100%, the (blackhole) attacker drops all received packets. If the attack rate is 50%, the (grayhole) attacker randomly drops half of the received data packets. If an attacker decides to forward a data packet, it simply transmits the packet to the next hop node (a relay node or the BS).

#### 5.1.4. Performance Evaluation Metrics

We compared the pure GRP, the beta GRP, and our proposed hybrid GRP in terms of the following performance evaluation metrics:

1. *Detection completion time (DCT)*: *DCT* is defined as the simulation time (seconds) when all attackers are detected by victim nodes whose packets are dropped by the attackers. *DCT* evaluates how quickly a trust model detects attackers.
2. *Detection rate (DR) and false alarm rate (FAR)*: As shown in Table 2, node *i* classifies node *j* into either *trustworthy* or *untrustworthy* based on node *j*’s trust value at node *i*. Node *i* may either correctly detect an attacker as an untrustworthy node (*Hit*) or fail in detecting it (*Miss*). *DR* is the probability that an attacker is being considered as an untrustworthy node. On the other hand, a trust model may misclassify a normal node (nonattacker) into an untrustworthy node (*False alarm*). We get *FAR* as the number of false alarms divided by the number of normal nodes, as defined in [36]. Unlike *DR* (0 ≤ *DR* ≤ 1), *FAR* can be over 1.0 since a node can be falsely detected multiple times by its neighbors. We use *DR* and *FAR* to evaluate the detection accuracy of a trust model.

3. *Packet delivery rate (PDR)*: *PDR* is the probability that a data packet is successfully delivered to the base station. A reliable, trust-based routing will deliver most of the data packets to the destination even in the presence of inside packet drop attackers in the network. We use *PDR* to evaluate the *reliability* of a trust-based routing.
4. *Total energy consumption (E_T_) and energy efficiency (e_S_)*: For the energy consumption analysis, we considered the total amount of energy consumed by transmitting and receiving data packets, *E_T_* (*J*). To get *E_T_*, we use the first order radio model [37]. In this model, the amount of energy required to send a *k* bit packet over distance *d*, *E_Tx_*, and to receive a *k* bit packet, *E_Rx_*, is:

(17) $$E_{Tx}\left(k,d\right)=E_{elec}\times k+E_{amp}\times k\times d^{2}$$

(18) $$E_{Rx}\left(k,d\right)=E_{elec}\times k$$

where *E_elec_* is the energy dissipation of the radio to run the transmitter and receiver circuitry and is 50 nJ/bit, and the transmit amplifier *E_amp_* is 100 pJ/bit/m^2^. By using Equations (17) and (18), we obtain the total energy consumption for data packet delivery *E_T_* by summing *E_Tx_* and *E_Rx_* for all nodes in the network. In addition, to see how efficiently a routing works in terms of energy, we used the energy efficiency *e_S_* (mJ) defined as:

(19) $$e_{S}=\frac{E_{T}}{N_{S}}$$

where *N_S_* is the total number of packets successfully delivered to the base station. The energy efficiency *e_S_* indicates the average energy consumption to successfully deliver one data packet to the destination.

5. *Other metrics* (*N_T_*, *N_A_*, and *N_NA_*): *N_T_* is the total number of data packets generated by all source nodes, *N_A_* is the total number of data packets dropped due to attacks, and *N_NA_* is the total number of packets dropped due to other network problems (non-attacks), such as collision or noise.

#### 5.1.5. OPNET Simulation Setup

**Figure 5:**
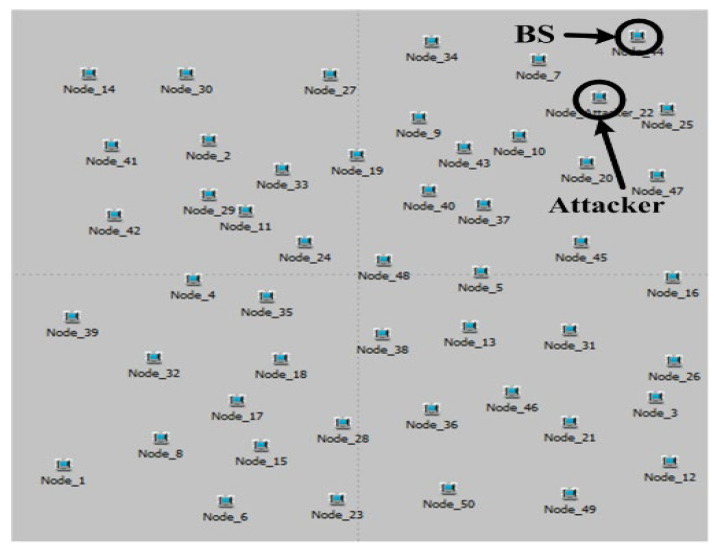
A random WSN topology in a 1 km × 1 km area.

The parameters in Table 3 are used in our simulations. An ad hoc WSN with 50 sensor nodes is built in a 1 km × 1 km area as shown in Figure 5. We place a single base station (BS) at one corner and every other sensor will generate and send data packets to the BS. We use the default OPNET setting of data packet size (1024 bits) and transmission power to 0.001 W. For the channel access model, we use CSMA/CA (carrier sense multiple access/collision avoidance) with a maximum of seven retransmissions (default value in OPNET). Each node generates a data packet randomly within each 10 s period as the simulation time of 100 s elapse, and forwards it toward the BS based on its routing algorithm, such as the pure GRP, the beta GRP, and our hybrid GRP. For trust models, we use three different trust thresholds of 0.6, 0.7, and 0.8. Considering a practical assumption that attackers develop a high trust value before launching attacks [31], we set the initial trust value of each node to be approximately 0.99. We place single or multiple attackers near the BS so that attackers can receive many data packets from nodes. Attackers start launching pre-defined attacks (blackhole attack or grayhole attack) once they receive data packets. By considering our simulation PC’s performance (Pentium CPU 3GHz and RAM 2 GB), we set the maximum simulation time to be three hours (10,800 s) to find the detection completion time *DCT*.

### 5.2. Simulation Results

We first compare three routing algorithms in the presence of the single attacker and multiple attackers in a WSN without burst transmission errors. Then, we compare them in a WSN with burst transmission errors.

#### 5.2.1. Single Attacker

Table 4 shows all simulation results in a single attacker case. All performance evaluation metrics in the first column are introduced in Section 5.1.4. When *DCT*s of both trust-based routings (beta GRP and our hybrid GRP) are found within the maximum simulation time (three hours), for comparison, we show the value of each metric measured at the slower *DCT*. When any trust-based routing algorithms fail to find *DCT* within the maximum simulation time, we show all metrics measured at the maximum simulation time. The pure GRP is compared to see how seriously the attacker degrades the network performance when we do not use any trust model.

We explain the results in detail in terms of detection speed, detection accuracy, routing reliability, and energy efficiency in turn.

(1) *Detection speed and the damage to the network*

**Figure 6:**
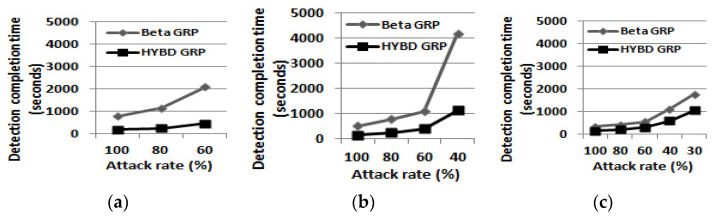
Detection completion time (DCT). (**a**) TH = 0.6. (**b**) TH = 0.7. (**c**) TH = 0.8.

To compare how *quickly* each trust model detects the inside packet drop attacker, we first examine the *DCT* of each routing algorithm as shown in Table 4 and Figure 6. When *DCT* is not found within three hours, we show *DR* instead; we will explain *DR* in detail in (*2*) *detection accuracy*. In general, when *TH* is fixed and as the attack rate grows, *DCT* decreases because the attacker drops more packets, its trust value decreases faster, and thus it is detected quicker by trust models. When the attack rate is fixed, as *TH* grows, *DCT* decreases because a trust model with a higher *TH* detects the attacker faster than the same trust model with a lower *TH*.

The results show the following:

First, our hybrid GRP can detect an inside packet drop attacker much faster than the beta GRP under various attack models, regardless of *TH*. When both trust models detect the attacker, our hybrid trust model can detect it at least 2.7 times and at most 4.7 times faster than the beta trust model. We found that when the attack rate is set to be 100 × (1 − *TH*), the beta trust model fails in detecting the attacker (i.e., *DCT* was not found) but our trust model can detect it. However, when the attack rate is set to be too low, both trust models failed in detecting the attacker.

**Figure 7:**
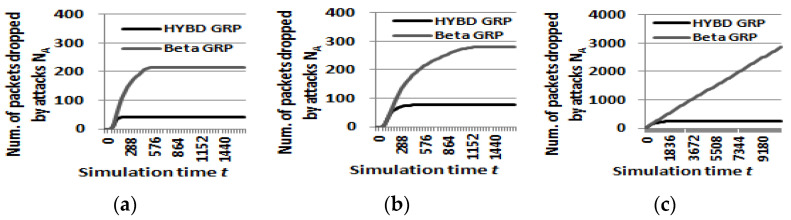
The number of packets dropped due to attacks N_A_ (TH = 0.7). (**a**) Attack rate = 100%. (**b**) Attack rate = 60%. (**c**) Attack rate = 30%.

Second, detection speed is closely related to the damage to the network caused by the attackers. The early detection capability of our hybrid trust model based on consecutive drops significantly reduces the damage to the network due to attacks compared to that of the beta trust model. In the case of the pure GRP, *N_A_* continuously grows as the simulation proceeds since it does not have a trust mechanism and thus cannot defend itself against the attacker. For the beta GRP and our hybrid GRP, when *DCT* is found, *N_A_* stops growing because the attacker is completely detected by all victim nodes (see Figure 7a,b). In addition, we can see the beta GRP’s *N_A_* is more than 3.5 times larger than our hybrid GRP’s *N_A_*. As shown in Figure 7c, when the beta GRP cannot find the attacker, *N_A_* grows continuously, and this degrades the performance of a routing as we will discuss in detail in (*3*) *reliability*.+

(2) *Detection accuracy*

In addition to early detection capability, a good trust model must have high detection accuracy, which can be evaluated by using *DR* and *FAR*. Thus, a desirable trust model has a high *DR* and a low *FAR*. A comparison of the detection accuracy of the beta trust model and our hybrid trust model is as follows.

**Figure 8:**
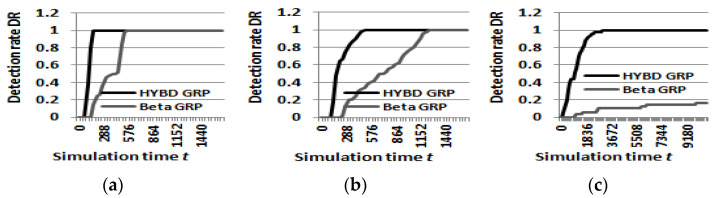
Detection rate DR (TH = 0.7). (**a**) Attack rate = 100%. (**b**) Attack rate = 60%. (**c**) Attack rate = 30%.

First, our hybrid trust model can detect an inside packet drop attacker not only more quickly, but also more accurately than the beta trust model. We already demonstrated that our hybrid trust model can detect the attacker much faster than the beta trust model in (*1*) *detection speed and the damage to the network.* Figure 8 shows how *DR*s of the beta GRP and our hybrid GRP change as the simulation time proceeds when *TH* is 0.7. In this figure, the value of (1 − *DR*) at time *t* indicates the percentage of victim nodes that cannot detect the attacker. That is, the difference between *DR*s of the two trust models shows the difference in the detection accuracy of the two trust models. The *DR* of our hybrid trust model is always above that of the beta trust model, which means that victim nodes within our hybrid trust model always detect the attacker faster and more accurately.

In particular, the beta trust model cannot completely detect the attacker when the attack rate is set to be equal to or less than 100 × (1 − *TH*). Meanwhile, our hybrid trust model can detect the attacker whose attack rate is 100 × (1 − *TH*). For example, in Figure 8c, when the attack rate is 30% and *TH* is 0.7, more than 80% of victim nodes with the beta trust model fail to detect the attacker within the maximum simulation time (three hours). Meanwhile, all victim nodes with our hybrid trust model detected the attacker in the early stage.

This is due to the limitation of the design of the beta trust model. That is, in the beta trust model, if an attacker continues to randomly drop 30% of received packets, its trust value *T* gradually lowers to 0.7 according to Equation (1). In practice, it is assumed that the attacker is likely to start dropping packets after it has a high trust value [31] to avoid being detected by a trust model. For this reason, the attacker’s trust value rarely falls below 0.7, and thus the beta trust model with *TH* = 0.7 fails to detect it. On the other hand, our hybrid trust model significantly penalizes the attacker’s repeating consecutive drops, and thus can detect the attacker with the same trust threshold. However, when the attack rate is less than 100 × (1 − *TH*), both the beta trust model and our hybrid trust model failed to detect the attacker. When the attacker knows the trust threshold, they can estimate its trust value before dropping any packet to make sure that they will never be caught. Although our hybrid trust model cannot defend against such an attacker, the damage that the attacker can cause to the network is limited according to the *TH*. For example, when the *TH* is 0.7, the attacker cannot drop more than 30% of packets without being caught. It will be interesting (and challenging) to study this problem, for example from the point of view of game theory [38,39]. However, since a detailed discussion to address this problem is out of the scope of this paper, we leave this for future work.

Second, our hybrid trust model generates a small *FAR* that is slightly higher than that of the beta trust model. A high *FAR* must be avoided, because it may negatively affect network connectivity, decrease the number of available relay nodes in the network, and thus significantly degrade the packet delivery performance of a routing. As shown in Table 4, we see that both our hybrid GRP and beta GRP do not have high *FAR*. Our hybrid GRP has slightly higher *FAR* than that of the beta GRP since our hybrid trust model gives a larger penalty to a node generating consecutive drops than the beta trust model. In practice, packets can be dropped consecutively due to some network problems as well as attacks. Therefore, this result was expected. In fact, as we will discuss below, this small *FAR* does not degrade the packet delivery performance of our hybrid GRP but helps to improve its packet delivery performance. Based on this result, we consider this small *FAR* acceptable to obtain high detection accuracy.

(3) *Routing reliability*

The final goal of a routing algorithm in a WSN is to deliver data packets from source nodes to the BS. Therefore, a good trust-based routing algorithm should be able to deliver packets to the destination successfully, even under insider packet drop attacks. We examine the routing reliability of each routing based on *PDR*. Recall that a single attacker is deployed near the BS to receive a significant number of data packets from many nodes throughout the network.

**Figure 9:**
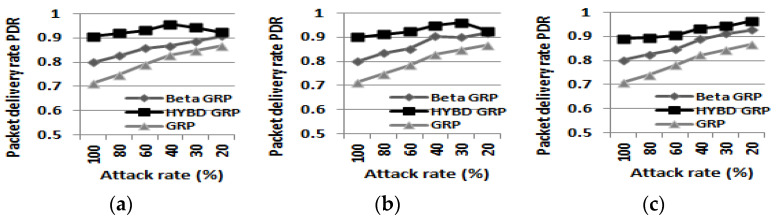
Packet delivery rate PDR. (**a**) TH = 0.6. (**b**) TH = 0.7. (**c**) TH = 0.8.

As shown in Table 4, our hybrid GRP has the highest *PDR* among the three routing algorithms and can deliver at least more than 89% of packets to the BS in all simulation setups. This result shows that our hybrid GRP can route packets very reliably even under various packet drop attacks. Specifically, our hybrid GRP improves the *PDR* of the pure GRP by at most 19% and the PDR of the beta GRP by at most 10% (when the attack rate is 100% and a *TH* of 0.6 is used, as shown in Figure 9a). Since *PDR* is determined by two metrics, *N_A_* and *N_NA_*, we examine them in turn.

First, as we explained in (*1*) *detection speed*, our hybrid GRP has the smallest *N_A_* among the three routing algorithms, as shown in Figure 7, because our hybrid GRP detects the attacker quickly based on consecutive drops and finds a new path to the BS through a trustworthy node. The beta GRP has a larger *N_A_* than our hybrid GRP because it has a slower detection speed than our hybrid GRP. Since the pure GRP does not use any trust model, it experiences the most significant amount of packet loss by the attacker.

**Figure 10:**
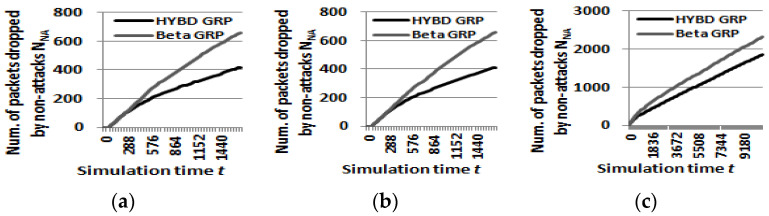
The number of packets dropped due to non-attacks N_NA_ (TH = 0.7). (**a**) Attack rate = 100%. (**b**) Attack rate = 60%. (**c**) Attack rate = 30%.

Second, our hybrid GRP has the least *N_NA_* among the three routing algorithms. Packets can be naturally dropped in a WSN for of many reasons, such as collision and congestion. The OPNET simulates channel quality problems, such as collision and congestion. As shown in Table 4, our hybrid GRP and the beta GRP have less *N_NA_* than that of the pure GRP. This is because a trust-based routing algorithm avoids bad communication links by classifying normal nodes (non-attackers) with many transmission errors as untrustworthy nodes. This causes both the beta GRP and our hybrid GRP to generate a small *FAR*, as previously discussed, but it improves the *PDR* of a trust-based routing algorithm. For the same reason as discussed in (*1*) *detection speed*, when normal nodes in bad links generate many consecutive drops, our hybrid GRP penalizes them based on the number of consecutive drops and thus quickly avoids them. As a result, our hybrid GRP has less *N_NA_* than the beta GRP, as shown in Figure 10. In practice, it is often assumed that WSNs have hundreds or thousands of sensors to be tolerant to many faulty sensors, so a small *FAR* may not be a problem.

(4) *Energy efficiency*

We compared the energy efficiency of each routing algorithm. The results show the following.

First, the total energy consumption of each routing algorithm is as follows: *E_T_* of our hybrid GRP > *E_T_* of the beta.

GRP > *E_T_* of the pure GRP as shown in Table 4. *E_T_* is closely related to *PDR*. In the pure GRP, a sender continues to forward data packets to a neighbor closest to the BS even though an attacker keeps dropping the packets. This is because the pure GRP has a mechanism to monitor neighbors’ packet transmission behaviors. The pure GRP has the least PDR, which means the largest amount of data packets are dropped before reaching the BS. Consequently, the pure GRP has the least amount of energy consumption for packet transmissions and receptions. Likewise, the beta GRP has less *E_T_* than our hybrid GRP. In addition, trust-based routings such as our hybrid GRP and the beta GRP are likely to have longer average hops or a longer average routing distance from a source to the BS than the pure GRP. This is because a trust-based routing avoids an inside packet drop attacker and normal nodes with many transmission errors and then finds another node. The avoided nodes are originally chosen as optimal nodes in terms of minimum hops; we note that the greedy geographic routing approximates the minimum hop routing [8], and thus we assume that the avoided node is likely to be in the shortest path to the BS. Since *E_T_* does not correctly evaluate the energy efficiency of a routing, we use the energy efficiency *e_S_* (mJ) for energy analysis.

**Figure 11:**
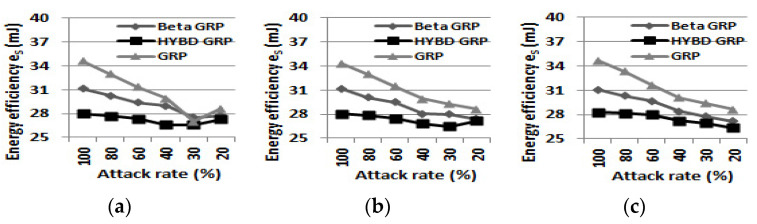
Energy efficiency e_S_. (**a**) TH = 0.6. (**b**) TH = 0.7. (**c**) TH = 0.8.

Second, our hybrid GRP consumes the least energy to deliver a packet to the BS among the three routing algorithms. As shown in Table 4, the energy efficiency *e_S_* of each routing is as follows: *e_S_* of pure GRP > *e_S_* of beta GRP > *e_S_* of hybird GRP. As shown in Figure 11, when the attack rate is 100% and TH is set to be 0.6, our hybrid GRP can save, at most, 20% more energy than the pure GRP and, at most, 10% more energy than the beta GRP, which corresponds to 6.61 mJ and 3.14 mJ, respectively. Using Equations (17) and (18) as an example, 6.61 mJ is the amount of energy that a node consumes to transmit a 1024-bit packet to another node that is approximately 253 m away.

#### 5.2.2. Multiple Attackers

We examine how each routing algorithm performs in the presence of multiple attackers. We assume that attackers are not colluding. To deploy multiple attackers in the network, we randomly choose 5, 8, and 10 relay nodes as attackers, which correspond to 5%, 10%, and 15% of total nodes, respectively. We use three attack rates of 30%, 60%, and 100%. For the trust threshold, we use 0.7. We compare all performance evaluation metrics recorded at the maximum simulation time (three hours). Since we thoroughly examined the detection speed of each routing in the single attacker case, we do not provide *DCT* here. In summary, the results in the multiple attacker case are similar with the results in the single attacker case. The results are as follows.

**Figure 12:**
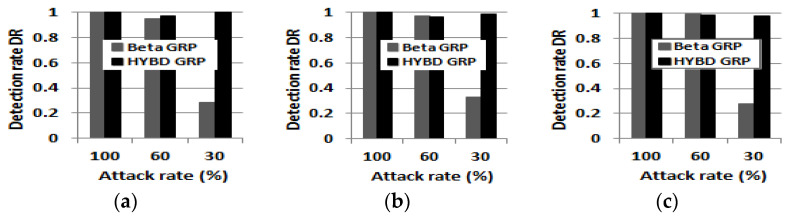
Detection rate DR (sultiple attackers). (**a**) 3 Attackers (5%). (**b**) 5 Attackers (10%). (**c**) 8 Attackers (15%).

**Figure 13:**
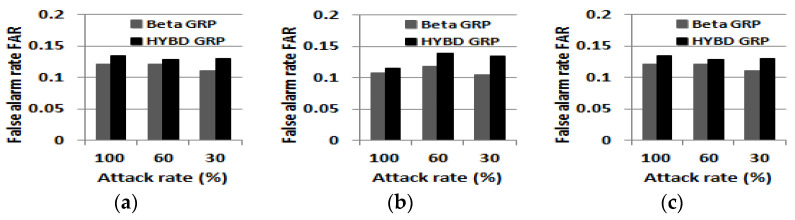
False alarm rate FAR (multiple attackers). (**a**) Three attackers (5%). (**b**) Five attackers (10%). (**c**) Eight attackers (15%).

First, we report *DR* and *FAR*. As in the single attacker case shown in Figure 12, our hybrid trust model based on consecutive drops has a very high *DR* in all simulation setups. Meanwhile, the beta GRP has a low *DR* in the presence of multiple grayhole attackers with a 30% attack rate. Our hybrid trust model also has a slightly higher *FAR* than the beta trust model (see Figure 13). However, as we will show regarding the *PDR* below, this small FAR (due to nodes in bad links) does not degrade the packet delivery performance of our hybrid GRP but improves the packet delivery performance of our hybrid GRP.

**Figure 14:**
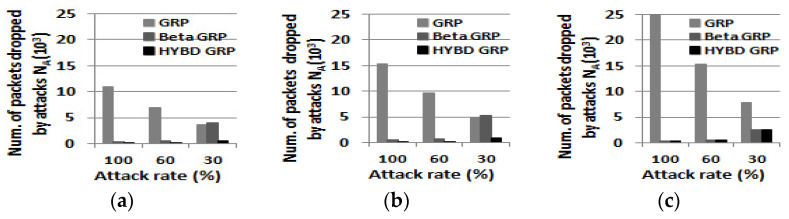
The number of packet drops due to attacks N_A_ (multiple attackers). (**a**) Three attackers (5%). (**b**) Five attackers (10%). (**c**) Eight attackers (15%).

**Figure 15:**
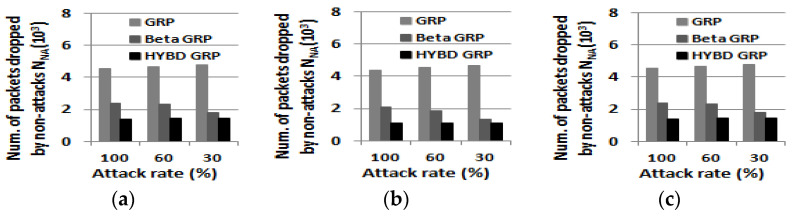
The number of packet drops due to non-attacks N_NA_ (multiple attackers). (**a**) Three attackers (5%). (**b**) Five attackers (10%). (**c**) Eight attackers (15%).

**Figure 16:**
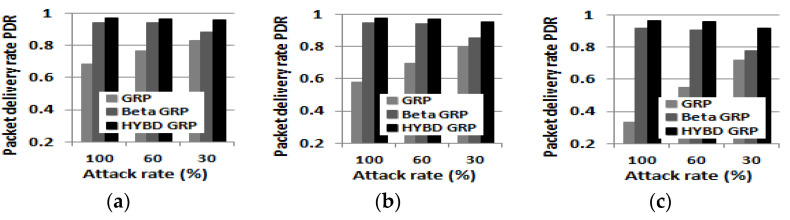
Packet delivery rate PDR (multiple attackers). (**a**) Three attackers (5%). (**b**) Five attackers (10%). (**c**) Eight attackers (15%).

Second, we consider *N_A_*, *N_NA_*, and *PDR*. In summary, as shown in Figure 14, Figure 15 and Figure 16, our hybrid GRP has the least *N_A_* and *N_NA_* and the highest *PDR* among the three routing algorithms in the presence of multiple attackers. As shown in Figure 14, it is not surprising that as the number of attackers grows, *N_A_* also grows. In the case of the pure GRP, we can see that multiple attackers (especially when they are blackhole attackers) drop a very large number of packets since the pure GRP does not have any defensive mechanism, such as a trust mechanism, against multiple attackers. Meanwhile, both our hybrid GRP and the beta GRP have much less *N_A_* than the pure GRP since their trust mechanisms can defend against the multiple attackers. In addition, thanks to the trust evaluation scheme using consecutive drops, our hybrid GRP is able to further decrease *N_A_* compared with the beta GRP. On the other hand, *N_NA_* does not significantly change as the number of attackers grows as shown in Figure 15. As in the single attacker case, our hybrid GRP has the least *N_NA_* because some normal nodes (non-attackers) with many transmission errors are detected (as false alarms) by our hybrid trust model. Finally, our hybrid GRP has the highest PDR, as shown in Figure 16. We clearly see that our hybrid GRP can deliver most packets to the BS very reliably, even in the presence of multiple attackers with various attack rates. It is particularly impressive that our hybrid GRP can deliver approximately 92% of data packets from source nodes to the BS successfully, even when 15% of nodes in the network are grayhole attackers that are randomly dropping 30% of packets, while the pure GRP and the beta GRP can deliver only 71% and 78% of data packets to the BS, respectively. In other words, when compared with the pure GRP, the beta GRP, which does not consider consecutive drops, can only improve *PDR* by 7%, but our hybrid GRP, which considers consecutive drops, can significantly improve *PDR* by 21%.

**Figure 17:**
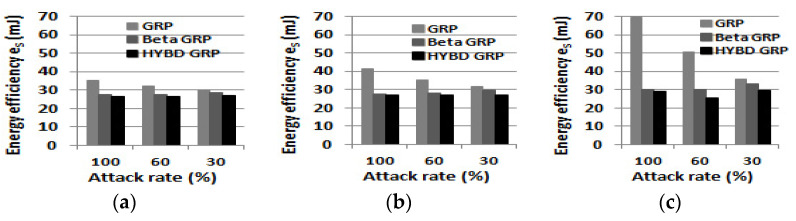
Energy efficiency e_S_ (multiple attackers). (**a**) Three attackers (5%). (**b**) Five attackers (10%). (**c**) Eight attackers (15%).

Finally, we report *e_S_*. Our hybrid GRP has the least *e_S_* among the three routing algorithms, as shown in Figure 17. As in the single attacker case, our hybrid GRP consumes the smallest amount of energy to deliver a data packet to the BS. In practice, the network operation time is much greater than our maximum simulation time (three hours). Therefore, when there are inside packet drop attackers in the network, the pure GRP and the beta GRP will waste a lot of energy on unsuccessful packet transmissions.

#### 5.2.3. Performance Comparison in a WSN with Temporal Burst Errors

In this section, we examine how our hybrid trust model works in a WSN where packets can be dropped consecutively due to fading or temporal obstacles as well as insider packet drop attacks.

We simulate this type of WSN in OPNET as follows. We deploy two blackhole attackers near the BS. In our simulation setup, 4% of packets are dropped naturally. We consider this natural packet drop rate as a normal error rate when there are no temporal errors in a WSN. Let the temporal error factor *k* be one at the normal error rate. To simulate temporal burst errors in a WSN, we temporarily increase the error rate by multiplying the normal error rate (=4%) by various *k* (up to 25) during a time duration (say, temporal error period). Then, during the period, packets are dropped with the increased error rate. We randomly choose 40% of nodes in which packets are dropped with various error rates during various error periods of 600, 1200, and 1800 s. The temporal error period starts randomly during the simulation. The higher the error rate and the longer the error period, the more packets are dropped during the error period; thus, the higher chance that packets are dropped consecutively.

We explain the results by using the two performance metrics of false alarm rate and packet drop rate.

First, our hybrid trust model does not generate a high false alarm rate, even in a WSN with various error rates and error periods. As shown in Table 5, in most setups (except when the error rate is extremely high), the false alarm rate of our hybrid trust model is the same as a normal error rate. This is because large consecutive drops are not likely to occur even when the temporal error rate is higher than the normal error rate. Meanwhile, when the error rate is set to be extremely high, such as 100%, the false alarm rate of our hybrid trust model is slightly increased to 0.11 because, in this setup, all packets are dropped consecutively. The false alarm rate of the beta trust model is increased to 0.02 when the temporal error period is 1800 s.

**Figure 18:**
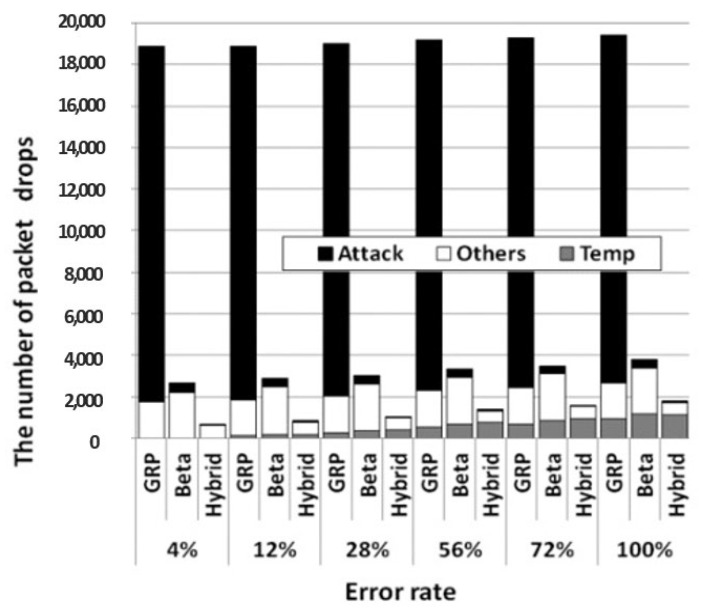
The number of packet drops at various error rates when the error period is set to be 1200 s.

Second, our hybrid trust model significantly reduces the packet drop rate of a routing, even in a WSN with various error rates and error periods. As shown in Table 5, when either the error rate or the error period grows, the packet drop rate of each routing also grows because packets are dropped more during the temporal error period. Figure 18 shows the detailed reasons for packet drops at various error rates when the error period is set to be 1200 s; in the figure, the number of packet drops due to attacks, temporal errors, and other network problems are depicted as attack (black), temp (gray), and others (white), respectively. When the pure GRP is used, most of packet drops occur due to the two undetected blackhole attackers. Meanwhile, since both trust-based routings can catch the attackers, they can dramatically reduce the packet drop rate compared to the pure GRP. In fact, our hybrid trust model avoids problematic nodes under the influence of temporal errors or collisions, as well as attackers, much faster than the beta trust model. Consequently, our hybrid trust model can further reduce packet drops.

## 6. Conclusions and Future Works

In this paper, we propose the use of consecutive drops for quick and accurate detection of inside packet drop attackers. We design a new trust model that probabilistically penalizes attackers based on the consecutive drops and build a hybrid trust model that adaptively uses the popular beta trust model and our new trust model. Extensive simulation results demonstrate that our hybrid trust model outperforms the beta trust model under various packet drop attacks in terms of detection speed, detection accuracy, routing reliability, and energy efficiency.

In our future work, we plan to study the following. First, we will study the distinct characteristics of colluding packet drop attackers and design defense mechanisms based on our hybrid trust model to defend against colluding packet drop attackers. Second, we will study applying the game theoretic approach for advancing our hybrid trust model to better defend against inside packet drop attacks. Third, we will study devising a new, sophisticated packet drop attack method avoiding the state-of-the-art defense methods, including our trust model. Finally, we will study an inside packet drop attack problem in a WSN adopting information fusion where intermediate nodes modify received packets, and thus existing trust mechanisms with the watchdog mechanism may not work because they cannot tell if a forwarded packet matches an observed packet.

## Figures and Tables

**Table 1:** Unit failure and unit penalty.

Unit Failure	Probability	Confidence	Confidence Ratio	Unit Penalty
Single f	*P*[*f*]	1 − *P*[*f*]	1(=(1 − *P*[*f*]^1^)/(1 − *P*[*f*]))	*α*
Double ff	*P*[*f*]^2^	1 − *P*[*f*]^2^	(1 − *P*[*f*]^2^)/(1 − *P*[*f*])	*α* × (1 − *P*[*f*]^2^)/(1 − *P*[*f*])
Triple fff	*P*[*f*]^3^	1 − *P*[*f*]^3^	(1 − *P*[*f*]^3^)/(1 − *P*[*f*])	*α* × (1 − *P*[*f*]^3^)/(1 − *P*[*f*])
*n*-tuple f…f	*P*[*f*]^n^	1 − *P*[*f*]^n^	(1 − *P*[*f*]^n^)/(1 − *P*[*f*])	*α* × (1 − *P*[*f*]^n^)/(1 − *P*[*f*])

**Table 2:** Detection and false alarm.

Given Node Is	Decision Result	
	Untrustworthy	Trustworthy
**Attacker**	Hit (Detection)	Miss
**Normal node**	False alarm	Correct rejection

**Table 3:** Simulation setup parameters.

Parameters		Setting
**General**	Terrain dimension	1 km × 1 km
	Number of nodes	50
	Topology	Random
	Max. simulation time	3 h (10,800 s)
	Number of runs	10
	Base routing algorithm	GRP
**Sensor**	Transmission power	0.001 W (OPNET default)
	Max. retransmissions	7 (OPNET default)
**Data packet** **Generation**	Start time–Stop time	100 s–end of simulation
	Destination	Base station
	Packet arrival interval	Every 10 s
	Packet size	1024 bits
**Trust model**	Type	None, Beta, and Hybrid
	Initial trust value	0.99
	Trust threshold (TH)	0.6, 0.7, and 0.8
**Attack model**	Number of attackers	Single attacker and multiple attackers (3, 5, 8)
	Attack type	Blackhole (attack rate 100%) and grayhole (attack rate 20–80%)

**Table 4:** Simulation results (single attacker). All performance evaluation metrics are shown in the first column.

Trust Threshold		TH = 0.6						TH = 0.7						TH = 0.8					
Attack Rate %		100	80	60	40	30	20	100	80	60	40	30	20	100	80	60	40	30	20
**DCT**	**Beta**	781	1155	2095	*0.04	*0.02	*0.02	522	774	1083	4190	*0.16	*0.02	342	424	558	1112	1771	*0.12
	**Hybrid**	183	244	457	1846	*0.88	*0.02	151	241	396	1123	2030	*0.02	151	212	295	597	1062	7376
**FAR**	**Beta**	0	0.037	0.039	0.097	0.104	0.097	0.002	0.037	0.045	0.125	0.114	0.112	0.014	0.039	0.043	0.104	0.118	0.141
	**Hybrid**	0.072	0.102	0.118	0.127	0.125	0.112	0.081	0.108	0.116	0.141	0.135	0.131	0.072	0.087	0.110	0.133	0.141	0.189
**N_A_**	**GRP**	661	820	1115	3883	2914	1894	427	534	595	1643	2914	1894	256	280	288	397	474	1894
	**Beta**	331	397	551	4176	3177	2078	213	236	278	580	2858	2113	123	128	139	183	240	2063
	**Hybrid**	44	64	96	265	989	2139	39	53	77	163	255	2134	29	42	57	84	120	432
**N_NA_**	**GRP**	336	509	946	4942	4931	4964	225	335	479	1975	4931	4964	141	182	243	508	838	4964
	**Beta**	364	515	858	2690	2702	2703	243	336	460	1190	2320	2118	148	183	231	375	482	1759
	**Hybrid**	282	364	591	1983	2002	1859	188	251	304	828	1853	1719	121	142	175	259	365	1474
**PDR**	**GRP**	0.711	0.747	0.790	0.828	0.847	0.866	0.712	0.747	0.784	0.828	0.847	0.866	0.708	0.739	0.779	0.822	0.843	0.866
	**Beta**	0.799	0.825	0.856	0.866	0.885	0.906	0.798	0.833	0.850	0.904	0.899	0.917	0.801	0.823	0.845	0.888	0.912	0.925
	**Hybrid**	0.906	0.918	0.929	0.956	0.941	0.922	0.899	0.911	0.922	0.947	0.958	0.925	0.890	0.894	0.903	0.932	0.941	0.962
**E_T_** **(J)**	**GRP**	84.9	129.8	242.1	1272.1	1272.9	1274.8	55.2	84.5	121.3	498.8	1272.9	1274.8	33.1	43.3	59.1	125.1	203.8	1274.8
	**Beta**	86.0	131.7	245.6	1290.2	1290.5	1292.9	56.0	86.0	123.2	511.7	1295.1	1291.7	33.7	43.8	60.0	127.8	208.4	1294.2
	**Hybrid**	88.0	133.9	248.7	1305.7	1306.0	1293.3	57.0	87.1	124.6	512.5	1304.7	1293.6	34.3	44.2	60.7	128.4	208.9	1304.9
**e_S_** **(mJ)**	**GRP**	34.61	32.98	31.34	29.88	26.97	28.63	34.29	32.95	31.48	29.87	29.26	28.63	34.63	33.31	31.66	30.06	29.35	28.63
	**Beta**	31.14	30.27	29.35	28.97	27.50	27.73	31.18	30.06	29.50	28.05	28.00	27.41	31.08	30.27	29.65	28.40	27.74	27.18
	**Hybrid**	28.00	27.67	27.35	26.57	26.57	27.29	28.06	27.86	27.48	26.80	26.49	27.19	28.28	28.17	27.97	27.20	26.95	26.35

* Detection rate (DR) is given when Detection Completion Time (DCT) is not found within the maximum simulation time.

**Table 5:** False alarm rate and packet drop rate in a WSN with temporal burst errors.

Error Period		600 s					1200 s					1800 s				
Error Rate %	*k*	FAR		Packet Drop Rate %			FAR		Packet Drop Rate %			FAR		Packet Drop Rate %		
		Beta	Hybrid	GRP	Beta	Hybrid	Beta	Hybrid	GRP	Beta	Hybrid	Beta	Hybrid	GRP	Beta	Hybrid
4	1	0	0.09	35.96	5.03	1.30	0	0.09	35.96	5.03	1.30	0	0.09	35.96	5.03	1.30
12	3	0	0.09	36.07	5.29	1.46	0	0.09	36.03	5.45	1.64	0	0.09	36.17	5.53	1.66
28	7	0	0.09	36.20	5.58	1.65	0	0.09	36.27	5.73	1.98	0	0.09	36.36	5.82	2.14
56	14	0	0.09	36.34	5.82	1.84	0	0.09	36.61	6.33	2.65	0	0.09	36.76	6.56	3.04
72	18	0	0.09	36.40	5.92	2.25	0	0.09	36.75	6.62	3.02	0.02	0.09	36.93	6.72	3.45
100	25	0	0.11	36.56	6.40	2.50	0	0.11	37.02	7.22	3.39	0.02	0.11	37.38	7.59	3.81

## Data Availability

Not applicable.

## References

[1] Djahel S., Nait-abdesselam F., Zhang Z. (2011). Mitigating Packet Dropping problem in Mobile Ad Hoc Networks: Proposals and Challenges. IEEE Trans. Surv. Tutor..

[2] Ishmanov F., Zikria Y.B. (2017). Trust Mechanisms to Secure Routing in Wireless Sensor Networks: Current State of the Research and Open Research Issues. J. Sens..

[3] Karlof C., Wagner D. (2003). Secure Routing in Wireless Sensor Networks: Attacks and Countermeasures. Ad Hoc Netw..

[4] Marti S., Giuli T.J., Lai K., Baker M. Mitigating Routing Misbehavior in Mobile and Ad Hoc Networks. Proceedings of the 6th Annual International Conference on Mobile Computing and Networking (Mobicom).

[5] Suh T., Cho Y. (2019). An Enhanced Trust Mechanism with Consensus-Based False Information Filtering Algorithm against Bad-Mouthing Attacks and False-Praise Attacks in WSNs. Electronics.

[6] Cho K., Cho Y. (2020). HyperLedger Fabric-Based Proactive Defense against Inside Attackers in the WSN With Trust Mechanism. Electronics.

[7] Sultana S., Bertino E., Shehab M. (2015). A Lightweight Secure Scheme for Detecting Provenance Forgery and Packet Drop Attacks in Wireless Sensor Networks. IEEE Trans. Dependable Secur. Comput..

[8] Sun Y.L., Yu W., Han Z., Liu K.J.R. (2006). Information Theoretic Framework of Trust Modeling and Evaluation for Ad Hoc Network. IEEE J. Sel. Areas Commun..

[9] Yu Y., Li K., Zhou W., Li P. (2012). Trust Mechanisms in Wireless Sensor Networks: Attack Analysis and Countermeasures. J. Netw. Comput. Appl..

[10] Zahariadis T., Leligou H.C., Trakadas P., Voliotis S. (2010). Trust Management in Wireless Sensor Networks. Eur. Trans. Telecommun..

[11] Pirzada A.A., McDonald C. Trusted Greedy Perimeter Stateless Routing. Proceedings of the 15th IEEE International Conference on Networks.

[12] Zahariadis T., Leligou H., Karkazis P., Trakadas P., Papaefstathiou I., Vangelatos C., Besson L. (2010). Design and Implementation of A Trust-aware Routing Protocol for Large WSNs. Int. J. Netw. Secur. Its Appl..

[13] Ahmed A., Bakar K.A., Channa M.I., Haseeb K., Khan A.W. (2015). TERP: A Trust and Energy Aware Routing Protocol for Wireless Sensor Network. IEEE Sens. J..

[14] Sun B., Li D. (2018). A Comprehensive Trust-Aware Routing Protocol ith Multi-Attributes for WSNs. IEEE Access.

[15] Hajar M.S., Al-Kadri M.O., Kalutarage H. LTMS: A Lightweight Trust Management System for Wireless Medical Sensor Networks. Proceedings of the 2020 IEEE 19th International Conference on Trust, Security and Privacy in Computing and Communications (TrustCom).

[16] Mehetre D.C., Roslin S.E., Wagh S.J. (2018). Detection and Prevention of black-hole and selective forwarding attack in clustered WSN with Active Trust. Clust. Comput..

[17] Cho J.-H., Swami A., Chen I.-R. (2011). A Survey on Trust Management for Mobile Ad Hoc Networks. IEEE Commun. Surv. Tutor..

[18] Josang A., Ismail R. The Beta Reputation System. Proceedings of the15th Bled Electronic Commerce.

[19] OPNET Modeler Wireless Suite. https://www.opnet.com/solutions/network_rd/modeler_wireless.html.

[20] Yu B., Xiao B. Detecting Selective Forwarding Attacks in Wireless Sensor Networks. Proceedings of the 20th IEEE International Parallel & Distributed Processing Symposium.

[21] Wang X.-S., Zhan Y.-Z., Xiong S.-M., Wang L.-M. Lightweight Defense Scheme against Selective Forwarding Attacks in Wireless Sensor Networks. Proceedings of the 2009 International Conference on Cyber-Enabled Distributed Computing and Knowledge Discovery.

[22] Hai T.H., Huh E.-N. Detecting Selective Forwarding Attacks in Wireless Sensor Networks Using Two-hops Neighbor Knowledge. Proceedings of the 2008 Seventh IEEE International Symposium on Network Computing and Applications.

[23] Khalil I., Bagchi S., Rotaru C.N., Shroff N.B. (2010). UnMask: Utilizing neighbor monitoring for attack mitigation in multihop wireless sensor networks. Ad Hoc Netw..

[24] Sun H.-M., Che C.-M., Hsiao Y.-C. An Efficient Countermeasure to the Selective Forwarding Attack in Wireless Sensor Network. Proceedings of the TENCON 2007—2007 IEEE Region 10 Conference.

[25] Navmani T.M., Yogesh P. (2019). Trust based Secure Reliable Route Discovery in Wireless Mesh Networks. KSII Trans. Internet Inf. Syst..

[26] Chen Z., Tian L., Lin C. (2017). Trust Model of Wireless Sensor Networks and Its Application in Data Fusio. Sensors.

[27] FIshmanov S., Kim W., Nam S.Y. (2015). A Robust Trust Establishment Scheme for Wireless Sensor Networks. Sensors.

[28] Casella G., Berger R.L. (1990). Statistical Inference.

[29] Karp B., Kung H.T. GPSR: Greedy Perimeter Stateless Routing for Wireless Networks. Proceedings of 6th Annual International Conference on Mobile Computing and Networking.

[30] Cho Y., Qu G., Wu Y. Insider Threats against Trust Mechanism with Watchdog and Defending Approaches in Wireless Sensor Networks. Proceedings of the 2012 IEEE Symposium on Security and Privacy Workshops.

[31] Lopez J., Roman R., Agudo I., Fernandez-Gago C. (2010). Trust management systems for wireless sensor networks: Best practices. Comput. Commun..

[32] McCune J.M., Shi E., Perrig A., Reiter M.K. Detection of Denial-of-Service Attacks on Sensor Network Broadcasts. Proceedings of the 2005 IEEE Symposium on Security and Privacy (S&P’05).

[33] Wood A.D., Stankovic J.A. (2002). Denial of Service in Sensor Networks. Computer.

[34] Zhang J., Li W., Yin Z., Liu S., Guo X. Forest Fire Detection System based on Wireless Sensor Network. Proceedings of the 2009 4th IEEE Conference on Industrial Electronics and Applications.

[35] Arampatzis T., Lygeros J., Manesis S. A Survey of Applications of Wireless Sensors and Wireless Sensor Networks. Proceedings of the 2005 IEEE International Symposium on, Mediterrean Conference on Control and Automation Intelligent Control.

[36] Sun Y., Han Z., Liu K.J.R. (2008). Defense of Trust Management Vulnerabilities in Distributed Networks. IEEE Commun. Mag..

[37] Heinzelman W.R., Chandrakasan A., Balakrishnan H. Energy-Efficient Communication Protocol for Wireless Microsensor Networks. Proceedings of the 33rd Annual Hawaii International Conference on System Sciences.

[38] Liang X., Xiao Y. (2013). Game Theory for Network Security. IEEE Commun. Surv. Tutor..

[39] Vijayalakshmi S., Bose S., Logeswari G., Anitha T. (2023). Hybrid defense mechanism against malicious packet dropping attack for MANET using game theory. Cyber Secur. Appl..

